# Behavior in Oblivion: The Neurobiology of Subliminal Priming

**DOI:** 10.3390/brainsci2020225

**Published:** 2012-05-29

**Authors:** Christianne Jacobs, Alexander T. Sack

**Affiliations:** 1 Department of Cognitive Neuroscience, FPN, Maastricht University, Maastricht, 6200 MD, The Netherlands; Email: a.sack@maastrichtuniversity.nl; 2 Maastricht Brain Imaging Center, M-BIC, Maastricht, 6200 MD, The Netherlands

**Keywords:** subliminal priming, neural correlates of consciousness (NCC), neural correlates of subliminal priming (NCSP), early visual cortex (EVC), visual awareness

## Abstract

Subliminal priming refers to behavioral modulation by an unconscious stimulus, and can thus be regarded as a form of unconscious visual processing. Theories on recurrent processing have suggested that the neural correlate of consciousness (NCC) comprises of the non-hierarchical transfer of stimulus-related information. According to these models, the neural correlate of subliminal priming (NCSP) corresponds to the visual processing within the feedforward sweep. Research from cognitive neuroscience on these two concepts and the relationship between them is discussed here. Evidence favoring the necessity of recurrent connectivity for visual awareness is accumulating, although some questions, such as the need for global *versus* local recurrent processing, are not clarified yet. However, this is not to say that recurrent processing is sufficient for consciousness, as a neural definition of consciousness in terms of recurrent connectivity would imply. We argue that the limited interest cognitive neuroscience currently has for the NCSP is undeserved, because the discovery of the NCSP can give insight into why people do (and do not) express certain behavior.

## 1. Introduction

During the 2000 U.S. Presidential elections, the Republican Party was accused of hiding a message in one of their television commercials. In the context of the Democratic Party’s policy, the word “rats” was flashed, presumably to establish a cognitive link associating Democrats with rats. But the presentation of the word was said to be so brief, that it could not be perceived consciously by viewers. Evidently, given that this commercial was broadcast on national television, rather than in a controlled laboratory environment, there are no data available on the extent to which people were indeed oblivious to the hidden message. However, much experimental research on the stimulus conditions modulating visual awareness has been performed, and it has been investigated whether any relevant stimulus-related information of these kinds of brief stimuli is processed, even if awareness is absent. So-called forced-choice paradigms require participants to judge a particular stimulus feature on each trial, also on trials when the participants claim no stimulus awareness. In this context, overall task performance can exceed the chance threshold [1,2], which indicates that relevant stimulus processing occurs, even if this does not suffice to instantiate conscious awareness. Because the stimulus does not cross the consciousness threshold (Greek *limen*), this form of perception is termed subliminal. Subliminal perception has inspired much research over the last decades, especially since the search for the neural correlates of consciousness (NCC) soared again in the 1990s. 

The concept of subliminal perception is fascinating, because it reflects the fact that our brains can “know” more than we consciously experience. This begs to question how our decisions are influenced by these unconscious (neural) processes. Going back to our everyday life example of political campaigning, we might ask ourselves the question: even if potential voters unconsciously perceived the hidden message, would this influence their voting behavior? Obviously, in the given example it would be very hard to determine, because of the vast amount of (confounding) variables which all contribute to people’s political preferences. But the basic question of the possible influence of a subliminal stimulus on subsequent behavior can be isolated and studied in a simplified laboratory environment. In psychology, priming, *i.e*., the facilitative or inhibitory effect on the behavioral response to a stimulus by a previously presented stimulus, is taken as a measure of behavioral impact. If the prime is rendered invisible, for example through visual masking, the behavioral response to the target stimulus can tell us in how far the prime is still processed. Priming is therefore an ideal manner of studying the behavioral influence of unconscious stimuli, and subliminal priming has indeed been applied in many psychophysical studies so far [3,4,5,6,7]. In this way, it could be demonstrated that political conviction and voting behavior are in fact modulated by subliminal stimuli, which were in this case subliminal images of the participants’ national flag [8]. 

Subliminal priming has also gained interest from a neuroscientific perspective, and next to the behavioral response, the neural response to subliminal stimuli has become a topic of scientific research. For an unconscious stimulus to steer future behavior, it must, at least to a certain extent, activate the neural machinery, which is in essence the generator of all behavior. The neural activity correlating with the priming capacity of a subliminal stimulus could be called the neural correlates of subliminal priming, or NCSP. On the other hand, the fact that the stimulus remains unaware, implies that a vital aspect of neural processing, namely the NCC, is missing. Tracing the neural signature of subliminal (and supraliminal) priming could bring us closer to solving the mystery of how the human brain creates consciousness.

**Figure 1:**
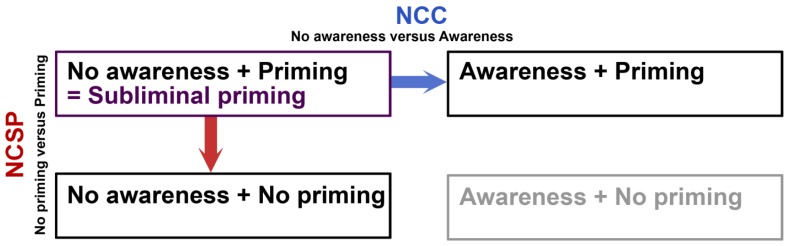
Subliminal perception in relation to NCC and NCSP. Subliminal priming is an interesting paradigm, because it entails the dissociation of visual awareness and behavioral impact, as measured by priming. Conditions of subliminal priming have mainly been compared to conditions of supraliminal priming (awareness and priming). This approach renders information about the neural correlates of consciousness (NCC). The opposite dimension reflects the contrast between subliminal priming and conditions in which the subliminal visual stimulus does not lead to priming. This approach can be informative with regards to the neural correlates of subliminal priming (NCSP). The fourth condition (awareness + no priming) is the situation that would theoretically complete the table, but it is implausible that awareness and priming could ever be actually dissociated like this and we therefore print it in gray here.

An approach that has been taken to identify the NCC is the subtraction of neural responses to aware *versus* unaware visual stimuli. The residual neural activity after subtraction is regarded to be part of the NCC. Lately, the idea has been put forward that the NCC consists of non-hierarchical recurrent (or reentrant) connections. The feedforward sweep, *i.e*., the ascending hierarchical processing stream, would be the corresponding NCSP. From this perspective, in order to influence behavior, a visual stimulus need only be processed in a feedforward manner. Analogous to the subtraction logic employed in search of the NCC, one could subtract the neural response to stimuli that evoke a behavioral response from those that do not and define the NCSP as that particular neural activity that survived this subtraction (see Figure 1). In reality, priming is greatly used in the search for an NCC, but relatively little systematic research has been done on the NCSP. This is unfortunate, because studying the NCSP in this way can lead to important insights as to what neural activity underlies the behavioral influence that environmental stimuli can have. 

In this review, we will discuss the role of recurrent connections for the NCC, and what the implications are for the NCSP. Studies employing functional neuroimaging and transcranial magnetic stimulation (TMS) have attempted to tear apart conscious vision and behavioral impact, and have gained important insights on the recurrent nature of the NCC. Nevertheless, we will argue that subliminal priming, rather than just being a tool to investigating the NCC deserves to be researched in its own right, because it might be even more fascinating to know what makes humans behave then to know what makes them conscious. 

## 2. Recurrent Processing and Visual Perception

All theories on NCC and NCSP that apply the principle of recurrent processing assume that the backprojection of information is the mechanism responsible for the constitution of awareness. These feedback processes take place following the initial feedforward sweep that transfers visual information hierarchically, from the lower levels of sensory cortex up to higher-level sensory areas and beyond to regions of the executive brain. In the visual domain the feedforward path is well-defined. After initial retinal processing, visual information travels through the lateral geniculate nucleus (LGN) of the thalamus and then enters the cortical brain in area V1, the primary visual cortex. Then, the information is parceled and information on specific visual features is conveyed to specialized processing units across the visual cortex. Some researchers believe that these higher level visual “modules” generate visual awareness [9]. In each specific processing unit, a local consciousness arises for the particular feature processed at that neural location. So, awareness of the color of a given stimulus would be generated in color processing area V4, whereas the motion awareness of the stimulus arises in hMT/V5. All these “microconsciousnesses” together make up the visual image we experience when seeing the stimulus. However, this hierarchical view on visual awareness has lost appeal over the last decade. Consensus has moved in the direction of recurrent processing as the mechanism underlying visual awareness [10].

Recurrent processing refers to the processing of information in a non-hierarchical fashion. Thus, if the neural signal no longer follows the road from low to high level brain regions, but instead takes a different horizontal or descending route, this is considered recurrent processing. Neuronal recordings in the macaque V1 have shown a modulation of late spiking responses supposed to reflect feedback activity from higher level visual cortex, which correlates with visual perception [11,12,13]. On the basis of these results, models of visual perception that assign a crucial role to recurrent connections have been proposed [14,15]. Similarly, the phenomenal aspect of vision, the experience of seeing a particular visual feature (“the redness of red”), has also been suggested to be caused by reverberation of the neural signal within the visual system. Even in the face of accumulating evidence acknowledging that recurrent processing is involved in many aspects of visual perception [11,16,17,18,19,20,21,22,23,24], the question remains whether recurrent activity is necessary for phenomenal awareness. In short, is recurrent processing (part of) the NCC? And there are related unresolved issues. First, there is still debate on the spatial scope of the relevant recurrent activity. As we will see later on, TMS studies point in the direction of local recurrent activity within sensory cortex as being functionally relevant to visual awareness. Alternatively, the relevant recurrent activity originates in executive regions of the fronto-parietal cortex, leading to a model of global recurrent interactions across the entire brain that is supported by neuroimaging data.

Second, what does this mean in relation to the NCSP? Possibly the unconscious processing of visual input is identical to conscious visual input, and the only difference is the absence of the NCC. Thus, if recurrent processing is taken to be the NCC, then all feedforward processing would reflect the neural correlate of the unconscious prime. However, it has also been suggested that unconscious vision is different in more ways, in the sense that the feedforward path of unconsciously processed visual input is already divergent. The signal that influences behavior might enter the cortex through a subcortical pathway circumventing V1, for example. However, data from TMS research are provided here that indicate that priming relies on a feedforward pathway via the early visual cortex (EVC) as well.

Finally, even if we decide that the necessity of recurrent connections for visual awareness has been empirically established, this is not to say that recurrent connectivity is also sufficient for awareness. If we take the approach of defining visual awareness in terms of recurrent processing, this implies that the presence of recurrent processing is sufficient to assign consciousness to someone (or something). This would have important implications for the research of state consciousness, and for the conclusions one draws from this type of research.

In the next sections, we will go over the currently held ideas on all of these matters and evaluate them based on recent neuroscientific research.

## 3. Global Recurrent Processing as NCC

The notion that something as complex as human phenomenal awareness would rely simply on processes in sensory brain regions, without any involvement of higher-level association regions in the fronto-parietal cortex, seems rather counterintuitive. Especially since psychophysical studies on semantic priming have shown that the visual information can be processed up to a high level [25]. The global neuronal workspace (GNW) model includes the fronto-parietal network in an extension of a model from cognitive psychology that considers focused attention to be a prerequisite for awareness [26]. According to both theories, there are two conditions that a stimulus needs to meet before it can gain access to conscious awareness. First, the stimulus needs to be of sufficient perceptual strength, and second, top-down focused attention needs to be directed towards it, which grants the information access to a so-called global workspace. In neural terms this means that processing information in sensory cortex is not enough [25,27,28]. Through the establishment of reverberating neuronal assemblies consisting of sensory neurons and “workspace” neurons in prefrontal and parietal brain regions, the information processed in sensory cortex becomes globally accessible. This idea is not new: Crick and Koch [29,30], the scientists that instigated new enthusiasm for consciousness in the neurosciences two decades ago, already proposed that for any information to reach awareness, it has to eventually be transferred to, and processed by, the prefrontal cortex. Nevertheless, they did not claim that the important connectivity between executive and sensory cortices should consist of recurrent loops. The GNW model makes some clear predictions when it comes to subliminal priming. If the physical stimulus is not strong enough or when focused attention is directed elsewhere, the information is not globally accessible, and the stimulus is thus not consciously perceived. But priming on the basis of this information can still occur, if the bottom-up strength of the prime stimulus is strong enough and it will initiate local neuronal synchrony [28]. Therefore, within the GNW framework, the strength of information processing restricted to the sensory cortex can be said to be the NCSP. 

Functional magnetic resonance imaging (fMRI) allows the visualization of task-related brain activation with high spatial resolution, but with relatively poor temporal resolution. Thus, fMRI is very valuable in assessing the exact location of neural processing, even if there is less temporal information in the signal. As the GNW model implies that fronto-parietal activity should be present when (visual) information is consciously perceived, fMRI is the optimal method for assessing the GNW, although valuable studies have been done using EEG (see e.g., [31]) and magnetoencephalography (MEG; see e.g., [32]). 

Dehaene *et al*. [33] found a left-lateralized network including the fronto-parietal and extrastriate visual areas to be activated to a larger extent for visible compared to invisible word stimuli, supporting the idea that for awareness generation to arise sensory information has to be accessible for a fronto-parietal neuronal workspace. In a later study, the neural correlates of supra- and subliminal orthographic priming were investigated with fMRI [34]. On trials when the prime was masked repetition suppression, *i.e*., the decrease in BOLD response as a consequence of repeated stimuli or stimulus properties, occurred in occipito-temporal cortex and frontal-eye fields. Despite its frontal location, the authors regarded the latter region as an early visual area, and concluded that the NCSP consists of processing in sensory, mainly occipito-temporal cortex. On the other hand, supraliminal primes lead to an even higher repetition suppression effect in occipito-temporal cortex (see also [35]), and additional effects in a fronto-parietal network. These results are in line with the idea that stronger visual stimuli will more easily activate the sensory modules, which in turn makes their information competitive for conscious accessibility through the GNW [28].

Unfortunately, correlational methods like MEG, EEG and fMRI do not permit claims about the necessity of the fronto-parietal cortex for visual awareness. A recent study found subjective awareness thresholds to be elevated in patients suffering from (left) prefrontal lesions, implying a causal role for prefrontal cortex in visual awareness [36]. Although lesion studies allow causal brain-behavior inferences, there are also numerous drawbacks, among which are the small patient numbers, the absence of pre-lesion data, and the brain’s ability to adapt itself to injury, so that the functional impact of the lesion is minimized. Transcranial magnetic stimulation (TMS) provides an alternative way of establishing causality by manipulation of brain activity in healthy individuals, avoiding the limitations associated with lesion patients. By the application of short-lasting transcranial magnetic pulses, which cause electric fields inside the brain and subsequent depolarization of neuronal axons, TMS allows non-invasive stimulation of cortical areas with high spatial and temporal specificity. Frontal TMS has been demonstrated to enhance detection and discrimination of visually presented stimuli [37,38] and to influence the voluntary switching of percepts in cases of ambiguous visual stimulation [39]. Target detection was similarly affected by TMS applied over parietal brain areas [40,41]. Furthermore, fMRI has shown that retinotopic occipital activation was affected by both frontal and parietal TMS [42,43], as well as by parietal brain lesion [44]. Together, these studies provide further support for the notion that fronto-parietal cortices are causally involved in the constitution of visual awareness. 

As mentioned before though, to confirm the claim that the bottom-up strength of the visual stimulus determines whether a stimulus has any behavioral impact, conditions of successful and unsuccessful priming should be compared, and should result in a higher activation amplitude in extrastriate areas during successful priming. The no-priming condition was not incorporated in the design of the described experiments and this crucial comparison therefore could not be made. 

Summarizing, we can conclude that the establishment of global recurrent loops of neural activity between the visual cortex and a network of fronto-parietal brain areas is probably (part of) the NCC, although it remains unclear whether the level of neural activity within the visual cortex contributes to behavioral modulation, and hence whether the proposed perceptual strength of the stimulus is (part of) the NCSP. 

## 4. TMS Studies in Favor of Local Recurrent Processing

Around the last turn of the century, the first theoretical models that implied feedback connections in the NCC were suggested [14,45,46,47,48]. At the same time a seminal paper brought about the first empirical evidence endorsing the view that recurrent processing is necessary for conscious visual perception [49]. Specifically, this study demonstrated the necessity of feedback from regions within extrastriate sensory cortex to V1 for visual awareness, and it could therefore be interpreted as supporting local recurrent processing as NCC. Pascual-Leone and Walsh (2001) [49] applied chronometric TMS, a technique that has been widely employed in the study of visual awareness, and that has been particularly utilized to investigate recurrent processing. There are three specific reasons why TMS is very suitable to study whether recurrent activity equals the NCC. 

First, because TMS offers high spatial and temporal specificity, the method can assess the involvement of neural activity at a given neural location, and at a given time point, for a given cognitive function. Applying single pulses of TMS to the occipital cortex around 90 ms post-stimulus onset can “mask” visual stimuli, as becomes evident from decreased task performance on visual discrimination tasks [49,50,51,52,53,54]. Thus, behavioral studies using visual masks can be mimicked with TMS in a so-called TMS-induced masking setting (see [55] for a comparison of visual masking and TMS masking results). 

Second, contrary to correlational neuroimaging methods such as fMRI or EEG, TMS can manipulate neural activity, and on the basis of TMS research one could thus conclude whether the recurrent activity, when present, actually bears any functional relevance to phenomenal experience. Obviously, this entails an experimental design that can isolate awareness from other visual processes, and consequently requires adequate selection of experimental and control tasks. 

A final advantage is the option to create with TMS phenomenal experiences of visual percepts in the absence of physical light stimulation; so-called phosphenes. Apart from the practical advantage that the position of a phosphene within the visual field can yield information about the corresponding retinal location which is affected by EVC stimulation, phosphenes are themselves a topic worthy of investigation. By using phosphenes as stimuli instead of physical stimuli, the neural signature of awareness can be studied without the need for feedforward activation of the visual system conveying information to the proper visual areas. 

Pascual-Leone and Walsh (2001) cleverly designed an experiment that makes optimal use of these advantages of TMS. They stimulated human visual motion area V5/hMT with single pulses of TMS, each of which induces visual awareness in the shape of moving phosphenes [49]. A second (subthreshold) TMS pulse was delivered over V1 to suppress the visual information processing in that area. The temporal delay between these two pulses was variable. Based on recurrency theory one would predict that the TMS-induced perceptual motion activity in V5 can only reach conscious awareness after the information has been backprojected to V1. The authors indeed found that the phosphene-related percept was impoverished if a TMS pulse was applied to V1 5–45 ms *after* V5 stimulation. Later, this result was replicated with real motion stimuli [56]. These studies demonstrate that feedback from motion area V5 to V1 is crucial for the conscious perception of motion, whether in response to physical or magnetic stimulation. 

Because of the temporal resolution in the millisecond range, TMS can also reveal the timing of feedforward and feedback processing in tasks of visual perception. In chronometric studies, single TMS pulses are applied across a broad range of time windows to find out at what time a cortical brain region is relevant to a particular task. The occipital TMS effect on visual awareness at multiple, distinct time windows has thus far been reported in several chronometric TMS studies employing different kinds of stimuli (e.g., [18,54,57,58]. The timings of such TMS effects are usually expressed in stimulus-onset-asynchrony, *i.e*., the difference in time between the onset of the visual stimulus and the TMS pulse. Ample studies have shown that TMS hampers visual perception when applied with a stimulus onset asynchrony (SOA) of ~90 ms. There is, however, debate as to whether this reflects the feedforward sweep or recurrent activity. Some have found an even earlier time window of TMS-induced masking, and therefore assume that it is feedback activity that TMS interferes with at 90 ms [58,59,60,61]. Others have discovered a later time window, and take the 90 ms time window to represent feedforward activity [18,22,62]. 

**Figure 2:**
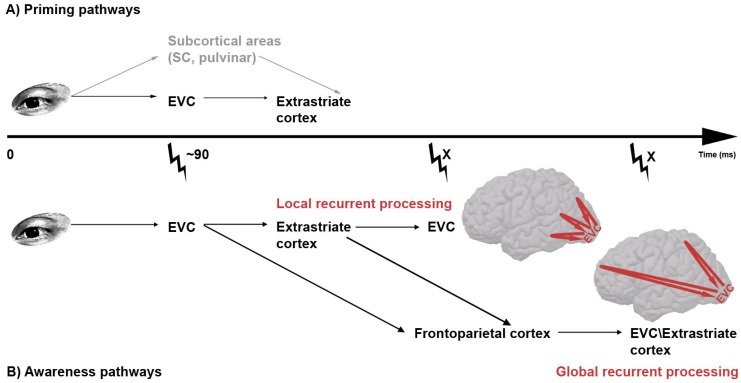
(**A**) Possible pathways of subliminal prime information. Two possible routes for unconscious visual prime processing have been suggested. In the top one (gray) the prime information travels from the eye via subcortical routes to extrastriate cortex, circumventing early visual cortex (EVC) altogether. In the lower route, prime information passes by EVC before further processing in extrastriate areas. The finding that transcranial magnetic stimulation (TMS; thunderbolt) applied to EVC 90 ms post-prime leads to decreased priming [53] renders support to the lower model; (**B**) Possible routes of supraliminal (conscious) visual stimulus. Two recurrent pathways for conscious visual stimulus processing have been suggested. Both pathways would predict that early visual cortex (EVC) is relevant for visual awareness at two time periods, the first reflecting feedforward processing and the second reflecting feedback. However, dependent on whether the origin of the feedback activity is placed in higher level areas *within* visual cortex (local recurrent processing) or *outside* visual cortex (global recurrent processing), the timing of this second period differs. Because there is empirical evidence in favor of each pathway, and because they are not mutually exclusive, we leave the possibility open that visual information can take both pathways.

Recently, a version of recurrent processing theory has been put forward that proposes local recurrent processing within EVC as opposed to global recurrent processing across more distant brain regions to be the process underlying visual awareness (see Figure 2B, [2,19]). Because of the emphasis on the local nature of the awareness-related neural modulation, the model predicts that it should only occur (tenths of) milliseconds after the feedforward sweep. Based on the observation that people report awareness of incorrectly bound features [19], they assume that the later global feedback activity is responsible for feature binding. The suggested time line of recurrent processing is supported by neurophysiological research [16,17]. MEG measures the magnetic field distributions, induced by the electrical activity of neuronal populations close to the scalp, over time. The magnetic physical properties allow accurate localization of the neural source with high temporal resolution. Boehler *et al*. [17] employed MEG in a study on visual awareness and discovered event-related modulations in V1 centered around 110 and 220 ms post-stimulus. Event-related activity in the first time window was found to correlate with visual awareness, and occurred only 11 ms after the first observations of feedforward responses in extrastriate areas. The much later modulation of neural responses in the striate cortex around 220 ms reflected attention-related recurrent processing. Evidently, the finding that early recurrent activity prior to the occurrence of attentional modulation correlated with visual awareness speaks in favor of local instead of global models of recurrent processing as NCC. 

De Graaf *et al*. [63] employed TMS pulses chronometrically to mask either orientation gratings or face stimuli. The first part of the classic TMS awareness dip around 100 ms proved identical for both stimulus types. In contrast, the recovery of the TMS masking function in the second half of the classic dip occurred faster for gratings than for faces, possibly because the information exchange with other brain areas is situated more locally for orientation gratings than for face stimuli. 

Summarizing, TMS studies on the NCC show that recurrent processing indeed appears to be functionally relevant for conscious visual awareness. Moreover, they hint particularly in the direction of local recurrent processing underlying visual awareness, which is contradictory to GNW theory. However, these results do not need to be mutually exclusive. The fMRI studies mentioned in the last paragraph use much more complex orthographic stimuli, which have a large semantic component and might therefore require feedback from higher level brain areas, whereas for the rather simple visual stimuli used in the here described TMS studies local recurrent processing might suffice. Recently, the stimulus-dependence of fronto-parietal involvement in visual awareness has already been suggested in the context of emotional stimulus processing [64].

Yet, to be certain that recurrent processing is the additional neural mechanism that can tear apart conscious from unconscious perception, and can serve as a marker for visual awareness as has sometimes been suggested [65] one needs to show that EVC TMS does not similarly affect behavior on a task that does not require any form of visual awareness, and therefore does not involve recurrent processing. Since we have seen that behavioral priming does not require phenomenal awareness of the prime stimulus, priming would be an optimal way to investigate whether the temporal dynamics of conscious and unconscious behavior are similar or different. 

## 5. The Road to Unconscious Vision

So far, we have seen that neuroscience has reasons to assume that recurrent connections are (part of) the NCC, although it is still an open question whether local sensory reentry is sufficient, or whether on top of this reentry from the executive fronto-parietal brain is necessary. The NCSP is linked to the processing of visual information in extrastriate areas, obviously in the absence of recurrent processing. But how does the visual information reach these extrastriate areas in the first place?

It has been suggested that the information that remains unconscious takes a different processing route that circumvents the primary visual cortex. Rather the information was proposed to travel either from LGN directly to extrastriate areas or to take a completely different route from the retina to superior colliculus and from there onwards to extrastriate regions [65]. Indeed, approximately 10 percent of visual input does not follow the classic retino-geniculate pathway. Further support comes from the neuropsychological phenomenon of blindsight. Due to a lesion in V1, blindsight patients experience a blind spot in their visual field, a spot where they do not experience any conscious awareness. However, when confronted with forced-choice paradigms, they can perform above-chance on a number of visual tasks, such as visual detection, localization, and motion perception [66], a finding which is as remarkable to the patients as to the experimenter, because subjectively they feel that they are simply guessing the correct answers. Next to subliminal priming, blindsight is another example of fairly adequate behavior driven by unconscious visual information. It seems logical to assume that if a stimulus can be processed unconsciously in the absence of intact EVC, an intact EVC is no prerequisite for unconscious information processing. From here, it follows that behavioral priming, a visual process that has been shown to not rely on conscious information processing [3,4,5,6,7], does not rely on an intact EVC either. Because TMS can induce a so-called “virtual lesion”, *i.e*., a temporal disruption of normal neural processing in healthy human individuals, we applied it to EVC to examine whether this idea indeed holds true. 

We employed a masked priming paradigm, in which we replaced the visual mask by a TMS pulse over EVC, the timing of which was systematically varied with SOAs ranging from 0 to 120 ms [53]. Visual recognition of the prime stimulus was disrupted with a single TMS pulse delivered 80 or 100 ms after visual presentation. However, the effect of the prime on a second stimulus also decreased by a TMS pulse delivered at these time points, suggesting that the neural activity required for priming was reliant on an intact retino-geniculo-striate pathway as well (see Figure 2A). A recent study has rendered further support for this conclusion. When the conscious awareness of a prime stimulus was completely abolished by a visual mask stimulus, as in a classical masked priming paradigm, EVC TMS proved to also interfere with priming [67].

In an alternative model of subliminal priming, the divergence between conscious and unconscious processing takes place at a later stage in the processing stream, namely at the moment when the visual signal starts to be fed back to lower neural levels. The bifurcation between unconscious and conscious information is very clear then: the feedforward sweep is unconscious, the feedback sweep is conscious. In that case, the classic 80–100 ms time windows at which we found our TMS effect here have to reflect feedforward activity, because priming is affected at these time windows as well. Consequently, there should be two time points at which TMS over early visual cortex would interfere with the recognition of a visual stimulus, the first one representing an interrupted feedforward sweep blocking any further (recurrent) processing of the visual stimulus, and a second one that represents the hindering of recurrent input into EVC, and that thus prevents the generation of a conscious percept. The priming effect of the same stimulus would only suffer from a TMS pulse at the early time window. After all, from psychophysical studies we know that no conscious awareness, and thus no feedback to EVC, is required for a stimulus to influence the behavioral response to a second stimulus [4,6,68,69]. 

Because of the limited temporal scope of our first TMS study, we decided to execute a follow-up study which tested a broader range of time windows with SOAs ranging from −80 to +300 ms in steps of 20 ms locked to prime-onset (Jacobs *et al*. [70]). Moreover, we included self-reported visual awareness as a subjective measure of visual awareness in order to rule out the possibility that the recognition task does not require any visual awareness, and that it is thus another measure of subliminal perception, rather than conscious vision. This did not prove to be the case, because, like task performance, self-reported awareness also decreased due to EVC TMS with an SOA of ~100 ms [62]. Comparing the temporal dynamics of visual discrimination and priming revealed a remarkably similar pattern. All three measures of visual perception, *i.e*., task performance, self-reported awareness and priming, suffered from TMS at similar SOAs. We replicated our earlier result, and again found EVC TMS to affect vision around 80–100 ms post prime onset. Moreover, the results revealed that pre-stimulus TMS, *i.e*., magnetic stimulation of EVC *prior* to the onset of the prime stimulus, could also hinder visual perception holistically. Contrary to what theories of recurrent processing predict though, no (late) TMS time window selectively impaired visual awareness. However, these data could be explained by the model of local recurrent processing [2,19]. According to this model, a second time window at which EVC TMS selectively hinders visual awareness, but not priming, would be separated from the classic (feedforward) time window by only milliseconds. Possibly, such a time window exists, but the resolution of our chronometric design was simply not high enough to detect it.

Summarizing, TMS-masked priming studies have shown that prime-related information, that does not require visual awareness, nevertheless takes the same feedforward route via EVC to extrastriate brain regions. The temporal dissociation between awareness and priming has not been established thus far, even though theories of recurrent processing would imply such a dissociation in the temporal domain. It remains to be seen whether future chronometric TMS research with an even higher temporal resolution will resolve this. 

Finally, we would like to stress that the models described above assume that conscious and unconscious visual perception are qualitatively different, and that they thus must have divergent processing pathways. In many of the studies described in this review, neural activity in response to visible *versus* invisible trials is compared, following the same discrete view on the nature of consciousness. An alternative approach would be to consider consciousness a dimensional phenomenon. From this perspective, binary response requirements, as in forced-choice paradigms, artificially create a dichotomous situation of “aware” *versus* “unaware” trials, which does not do justice to the true nature of visual awareness. Contrasting neural activity of both conditions then ignores the residual awareness potentially present in trials classified as “unaware”. It has therefore been suggested to additionally acquire subjective visibility ratings or confidence ratings on a multiple-point scale, and to correlate these scores with measures of accuracy and neural activity [64,71]. 

## 6. Implications and Future Research

Recurrent connections have been implicated in many aspects of visual perception, not the least of which is the phenomenal awareness of visual stimuli. The neuroscientific evidence that recurrent connections are (part of) the NCC is accumulating. But for some researchers this does not go far enough. Victor Lamme, one of the earliest and most visible proponents of recurrent connections as the NCC, has suggested that we no longer speak of the neural correlate of consciousness, but rather of a neural definition of consciousness (NDC [65]). Not surprisingly, he states that defining consciousness in terms of recurrent connectivity makes most sense. One might wonder whether the time is ripe for such a paradigmatic change. Even though empirical evidence implying recurrent activity in awareness is increasing, this does not automatically lead to the conclusion that the recurrent activity suffices for awareness, a conclusion which is implicitly part of the NDC concept. 

Defining consciousness in terms of recurrent activity could have far-stretched consequences, for instance in the field of state consciousness. A person could be declared to be in a certain state of consciousness purely based on the presence or absence of recurrent activity. The reversed approach has been applied. People with the clinical diagnosis of being in vegetative state were shown to have intact feedforward but impaired feedback processing based on their EEG responses [72]. Perhaps the interesting finding here is the preserved feedforward activation, rather than lack of recurrent processing. In an earlier study, vegetative state patients’ fMRI activity was compared to that of healthy observers after the instruction to visualize certain actions [73]. The pattern of brain activity proved to be very similar across the two groups. The authors concluded that vegetative state patients might actually experience a form of awareness. These two studies immediately make clear that there is a certain circularity of reasoning in the research on state consciousness, as the first one takes the absence of awareness as a given and investigates the accompanying brain activity to make statements about the NCC, whereas the second takes the neural activity as a starting point to make claims about the presence of awareness in vegetative state patients. By defining consciousness in terms of recurrent projections, this issue is superficially solved simply by choosing in favor of the latter directionality. 

But there are more differences between vegetative state patients and healthy humans. The most obvious is the lack of sensible stimulus-response behavior. As subliminal priming studies have shown, visual awareness and behavioral influence can operate independently. Why is it that the (feedforward) brain activity that neuroscience finds in vegetative state patients does not even lead to the simplest kinds of behavior? The discovery of the NCSP could be extremely insightful here, and the quest for it much more rewarding. After all, we can objectively assess the presence or absence of meaningful behavior, something which we cannot and will never achieve with regards to consciousness. Not only does this apply to patients, but in principle to all other individuals. When investigating the NCSP we avoid circling the same loop that researchers of the NCC are in at the moment. To know why people behave the way they do is the fundamental issue any cognitive science would be inclined to solve. Moreover, it is a question that is answerable.
